# Deep learning versus iterative image reconstruction algorithm for head CT in trauma

**DOI:** 10.1007/s10140-021-02012-2

**Published:** 2022-01-05

**Authors:** Zlatan Alagic, Jacqueline Diaz Cardenas, Kolbeinn Halldorsson, Vitali Grozman, Stig Wallgren, Chikako Suzuki, Johan Helmenkamp, Seppo K. Koskinen

**Affiliations:** 1 Department of Diagnostic Radiology, Karolinska University Hospital, 171 76 Stockholm, Sweden; 2 Department of Clinical Science, Intervention and Technology (CLINTEC), Karolinska Institutet, 17177 Stockholm, Sweden; 3 Department of Molecular Medicine and Surgery, Karolinska Institutet, 17177 Stockholm, Sweden; 4 Department of Medical Physics and Nuclear Medicine, Karolinska University Hospital, 17176 Stockholm, Sweden

**Keywords:** Image quality, Trauma, Head CT, Deep learning image reconstruction, Adaptive statistical iterative reconstruction-Veo

## Abstract

**Purpose:**

To compare the image quality between a deep learning–based image reconstruction algorithm (DLIR) and an adaptive statistical iterative reconstruction algorithm (ASiR-V) in noncontrast trauma head CT.

**Methods:**

Head CT scans from 94 consecutive trauma patients were included. Images were reconstructed with ASiR-V 50% and the DLIR strengths: low (DLIR-L), medium (DLIR-M), and high (DLIR-H). The image quality was assessed quantitatively and qualitatively and compared between the different reconstruction algorithms. Inter-reader agreement was assessed by weighted kappa.

**Results:**

DLIR-M and DLIR-H demonstrated lower image noise (*p* < 0.001 for all pairwise comparisons), higher SNR of up to 82.9% (*p* < 0.001), and higher CNR of up to 53.3% (*p* < 0.001) compared to ASiR-V. DLIR-H outperformed other DLIR strengths (*p* ranging from < 0.001 to 0.016). DLIR-M outperformed DLIR-L (*p* < 0.001) and ASiR-V (*p* < 0.001). The distribution of reader scores for DLIR-M and DLIR-H shifted towards higher scores compared to DLIR-L and ASiR-V. There was a tendency towards higher scores with increasing DLIR strengths. There were fewer non-diagnostic CT series for DLIR-M and DLIR-H compared to ASiR-V and DLIR-L. No images were graded as non-diagnostic for DLIR-H regarding intracranial hemorrhage. The inter-reader agreement was fair-good between the second most and the less experienced reader, poor-moderate between the most and the less experienced reader, and poor-fair between the most and the second most experienced reader.

**Conclusion:**

The image quality of trauma head CT series reconstructed with DLIR outperformed those reconstructed with ASiR-V. In particular, DLIR-M and DLIR-H demonstrated significantly improved image quality and fewer non-diagnostic images. The improvement in qualitative image quality was greater for the second most and the less experienced readers compared to the most experienced reader.

**Supplementary Information:**

The online version contains supplementary material available at 10.1007/s10140-021-02012-2.

## Introduction

There is wide consensus that noncontrast head CT is the initial imaging modality of choice for acute moderate to severe traumatic brain injury. CT is specific and sensitive for detecting intracranial hemorrhage (ICH) [1]. However, certain anatomical regions such as the middle and the posterior fossa are more prone to beam hardening artifacts from adjacent dense skull structures which can obscure subtle traumatic lesions [2–4]. Furthermore, CT without intravenous contrast agents has intrinsically relatively limited soft tissue contrast resolution [5, 6], e.g., in the brain parenchyma where the difference in CT attenuation between gray and white matter is relatively small. The diagnosis of some intracranial pathologies relies on the detection of the discreet alteration in attenuation that they cause between the gray and white matter [5]. These factors can make the interpretation of noncontrast trauma head CT challenging.

Iterative reconstruction (IR) techniques have demonstrated superior image quality for head CT compared to filtered back projection (FBP) with reductions in artifacts and image noise [7–10]. One reported disadvantage of IR compared to FBP is that its noise reduction can cause a blotchy or “pixelated” image texture [11–13] which is more pronounced with higher IR strengths [14–16]. Furthermore, studies have shown that IR achieves inferior resolution compared to FBP for low-contrast features [17], especially at low doses [18, 19].

Artificial intelligence (AI) has in recent years unlocked new possibilities in medical imaging including new methods for CT image reconstruction where deep learning (DL), a subset of machine learning, has been implemented [20]. DL is based on an artificial neural network inspired by the human brain in the sense that it automatically and unsupervised learns distinctive features from the input data itself which gives DL the ability to estimate highly complex nonlinear relationships. The term “deep” refers to the multilayer networks of artificial neurons [20, 21].

The DL-based image reconstruction (DLIR) algorithm TrueFidelity has recently been introduced by GE Healthcare. It has been trained on ground truth data comprising CT images reconstructed with FBP from a great number of phantom and patient cases including different anatomies, clinical indications, and scanning conditions. Both a low-dose/high-noise and high-dose/low-noise dataset was obtained for each case and the latter was used as the ground truth data. The DLIR algorithm was applied on the low-dose/high-noise datasets with the high-dose/low-noise datasets being the training target. CT image quality experts from GE Healthcare and radiologists supervised the training process. TrueFidelity offers three selectable strength levels (low, medium, and high) [22].

As novel image reconstruction algorithms are put on the market, it is important to conduct an independent assessment of their effect on image quality. Recent studies have shown that DLIR algorithms improve head CT image quality compared to IR [23–25]. However, none of these studies has evaluated how a DLIR algorithm performs compared to an IR algorithm on noncontrast head CT from a cohort of multitrauma patients presenting to a level 1 trauma unit. Our hypothesis is that DLIR will improve the conspicuity of trauma-related findings by decreasing the beam-hardening artifacts and reducing the image noise level, compared to IR. Hence, the purpose of this study was to evaluate the qualitative and quantitative image quality of noncontrast trauma head CT reconstructed with TrueFidelity with all three DLIR strengths and compare it to the IR algorithm adaptive statistical iterative reconstruction-Veo (ASiR-V), also by GE Healthcare.

## Materials and methods

This study has been approved by the regional ethics committee (Dnr 2020–04700, 2021–02547).

### Patient population

From 27 September 2020 until 15 December 2020, we retrospectively included 98 consecutive trauma patients who underwent a noncontrast head CT as a part of their trauma CT protocol at the level 1 trauma unit at Karolinska University Hospital, Stockholm, Sweden. Four patients were excluded. Three because the CT series were used as educational cases during the training of the readers, and one because the CT series were incomplete. Finally, a total of 94 patients were included in the study.

### CT protocol

The trauma CT scans were performed using a 512-slice scanner (Revolution CT, GE.

Healthcare). The following imaging parameters were used: scan mode, axial; scan field of view, head; display field of view, 230 mm; tube potential, 120 kV; tube current, 290 mA; detector coverage, 100, 100 mm (Smart Coverage); rotation time, 1.0 s; number of rotations per scan, 2; slice thickness, 0.625 mm; slice overlap, 0.3125 mm (only available for ASiR-V); default scan length, 180 mm (can vary depending on patient size); filter, standard.

Images were reconstructed with ASiR-V 50% and all three levels of TrueFidelity. Hence, we obtained four image series for each patient: ASiR-V 50%, TrueFidelity low (DLIR-L), medium (DLIR-M), and high (DLIR-H) strength. Hence, a total of 376 (4 × 94) noncontrast head CT series were evaluated.

### Quantitative image quality analysis

Images were evaluated on a PACS workstation (Sectra PACS IDS7, v.21.1, Linköping, Sweden). One radiologist with 7 years’ experience of trauma radiology (AZ), who was not involved in the qualitative image quality analysis, performed the quantitative image analysis.

The following five parameters were assessed: (1) CT attenuation of gray and white matter; (2) noise measurement of air, gray matter, white matter, and where applicable in the ICH; (3) signal-to-noise ratio (SNR) in the gray matter, white matter, and where applicable in the ICH; (4) gray-white matter differentiation; and (5) artifacts in the posterior cranial fossa. CT attenuation was defined as the mean Hounsfield unit (HU) values and the noise as the standard deviation (SD) of HU values.

Circular equal regions of interest (ROIs) were placed at the basal ganglia level in the right thalamus and in the right posterior limb of the internal capsule (PLIC). The thalamus was selected to represent the deep gray matter in our study because it enabled uniform ROI measurements. The head of the caudate nucleus was not chosen for this purpose because it is smaller than the thalamus. The lentiform nucleus was not chosen either because the globus pallidus has a higher myelin content compared to the putamen. ROIs were also placed in the right M5 cortex region (lateral MCA territory, equivalent to the Alberta Stroke Program Early CT Score — ASPECTS [26]) and in the adjacent white matter of the centrum semiovale (CSO), to maintain uniformity. In cases where obvious neuropathological CT findings were present, measurements in the corresponding structures of the contralateral hemisphere were performed. The ROIs measured between 4 and 6 mm in diameter for the thalamus and for the white matter, and 2 and 3 mm for the cortical gray matter, with minor size adjustments between patients to prevent volume averaging with nearby structures of different attenuation. For each ROI, the mean HU value and the SD of HU values were measured. The SNR was determined by the formula $\text{SNR = mean HU}/{\text{SD}}$

The noise measurement of air was performed by placing a 10-mm ROI in the air surrounding the study object at the level of the basal ganglia and measuring the SD of HU values. In cases where one or several different types of ICHs were present, a ROI measuring between 3 and 6 mm was placed in a homogenous part of each ICH type and the mean HU value as well as the SD of HU values were measured.

The gray-white matter differentiation was assessed by calculating the contrast-to-noise ratio (CNR) between the thalamic gray matter and the white matter of the PLIC as well as between the M5 cortex and the adjacent white matter of the CSO, respectively. The following formula was used: $\mathrm{CNR}=\frac{({\mathrm{mean HU}}_{\mathrm{Gray matter }}- {\mathrm{mean HU}}_{\mathrm{White matter})}}{\left({\mathrm{SD}}_{\mathrm{Gray matter}}+ {\mathrm{SD}}_{\mathrm{White matter}}\right) \times 0.5}$

The artifact evaluation in the posterior cranial fossa was assessed by placing a 15–17-mm ROI in the interpetrous region of the pons on the section with the most prominent artifacts. The SD of HU values within the ROI (image noise) was used as a surrogate measure for artifacts, as described by Kim et al. [23].

### Qualitative image quality analysis

The qualitative image quality was evaluated by three readers independently, comprising two radiologists with a board certificate recognized in the European Union: WS with 32 years’ experience (most experienced) and GV with 10 years’ experience (second most experienced) of trauma radiology; and one radiology resident: HK with 1.5 years’ experience (less experienced) of trauma radiology. Each reader was blinded to who the other readers were. The readers were also blinded to each other’s evaluations, to which of the four reconstructions that was applied to which CT series, and to the results from the quantitative image quality analysis. Prior to the readers’ assessment, each reader independently received a 1-h training session including a presentation of educational trauma cases with accompanying image quality scores that were previously reached by consensus of three experienced radiologists (KSK, SC, and AZ). These educational cases were excluded from the study. The CT series were anonymized and displayed next to each other in a random order. The CT series comprised 0.625-mm-thick slices and were presented with the multiplanar reconstruction function of the PACS. The readers assessed the following four parameters: noise, brain structures, artifacts, and ICH conspicuity. The scoring system (see Table 1) was based on the European guidelines on quality criteria for CT [27]. Scores could be used more than once if reconstructions were deemed equivalent.

**Table 1:** Scoring system for the evaluation of the qualitative image quality parameters

Parameter	Score				
	1	2	3	4	5
Image noise	Very noisy, non-diagnostic image quality	Noisy, but permits evaluation	Moderate noise	Low noise	Little to no noise
Brain structures	Very blurry, white/gray matter border, basal ganglia, CSF-spaces cannot be delineated, non-diagnostic	Blurry, white/gray matter border, basal ganglia, CSF-spaces can be delineated, permits evaluation	Moderate sharpness of white/gray matter border, basal ganglia, CSF-spaces	Good sharpness of white/gray matter border, basal ganglia, CSF-spaces	Sharpest visualization of white/gray matter border, basal ganglia, CSF-spaces
Artifacts	Very severe artifacts, non-diagnostic image quality	Severe artifacts, permits evaluation	Moderate artifacts	Mild artifacts	No artifacts
Intracranial hemorrhage conspicuity	Poor image quality, ICH is not displayed at all	Weak image quality, ICH is not well displayed and the image quality is insufficient for diagnosis	Satisfactory image quality, ICH is not clearly displayed but sufficiently enough to make a diagnosis	Good image quality, ICH is well displayed and can be diagnosed	Excellent image quality, ICH is very well displayed and easy to diagnose

*ICH* intracranial hemorrhage

The location and type of ICH was retrieved from the original trauma CT report, verified by AZ, and incorporated into the patient’s unique evaluation table so that all three readers graded the exact same ICH. If a patient had more than one ICH type, each type was graded separately.

### Statistical analysis

IBM SPSS (v.27, Chicago, IL, USA) was used to perform the statistical analyses. Continuous variables were reported as mean ± SD and ordinal variables as percentages. Two-sided significance level was set to 0.05.

Continuous variables, comprising quantitative image quality parameters, were compared between the four groups (ASiR-V, DLIR-L, DLIR-M, and DLIR-H). Means were compared with a one-way repeated-measures analysis of variance (ANOVA), and if assumptions were violated, the Friedman test was used instead.

Ordinal variables, comprising qualitative image quality scores, were compared between the four groups with the Friedman test.

Following a statistically significant repeated-measures ANOVA, post hoc pairwise comparisons were performed with paired *t*-tests and *p*-values were adjusted with the Bonferroni correction. Following a statistically significant Friedman test, post hoc pairwise comparisons were performed with the Dunn-Bonferroni test.

Normality testing was performed using the Shapiro-Wilks test. Mauchly’s sphericity test was used to validate the repeated-measures ANOVA regarding the sphericity assumption.

Weighted kappa (*κ*_w_) with linear weights was used to evaluate the inter-reader agreement. All *κ*_w_-values were interpreted according to Altman [28]: < 0.20 poor agreement, 0.21–0.40 fair agreement, 0.41–0.60 moderate agreement, 0.61–0.80 good agreement, and 0.81–1.00 very good agreement.

## Results

### Patient demographics, intracranial hemorrhages, and radiation dose

The study population comprised 70 males and 24 females with a mean age of 42.0 ± 20.4 years.

A total of 28 ICHs in 13 patients (13.8%; 13/94) were diagnosed, comprising seven contusions/intracerebral (25.0%), 12 subarachnoid (42.9%), six subdural (21.4%), and three epidural (10.7%) hemorrhages. Trauma mechanisms comprised four falls (30.8%), one assault (7.7%), three pedestrian-vehicle accidents (23.1%), two bicycle accidents (15.4%), one motor vehicle accident (7.7%), and two non-trauma-related (spontaneous intracerebral hemorrhages) (15.4%).

The mean volume CT dose index (CTDIvol) was 46.96 ± 0.49 mGy and the mean dose-length product (DLP) was 847.84 ± 22.25 mGy ∗ cm.

### Quantitative image quality analysis

**Fig. 1:**
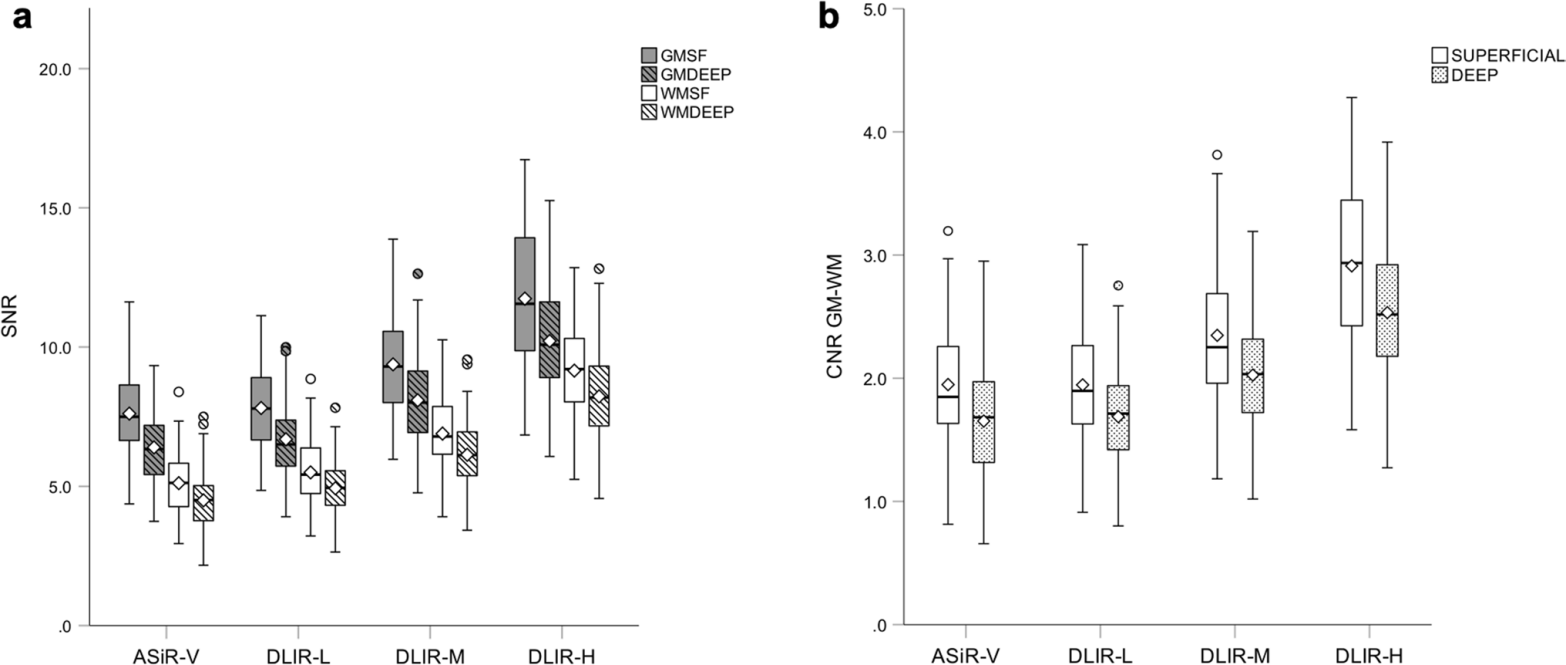
Clustered boxplot diagrams showing **a** SNR for superficial gray matter (GMSF), deep gray matter (GMDEEP), superficial white matter (WMSF), and deep white matter (WMDEEP) and **b** CNR between superficial gray and white matter, and deep gray and white matter, for ASiR-V and all three DLIR reconstruction strengths. For SNR, there was a statistically significant difference between all reconstructions, except between ASiR-V and DLIR-L for the deep and the superficial gray matter. For CNR, there was a statistically significant difference in mean CNR between all reconstructions, except between ASiR-V and DLIR-L. Both SNR and CNR gradually increased with higher DLIR strength levels. *Boxes* represent the middle 50% of the data, *solid lines* represent the median, and *white diamonds* represent the mean. The *whiskers* represent the minimum and maximum values. *Circles* represent outliers

The results from the quantitative image quality analysis are summarized in Table 2 and Fig. 1. The post hoc pairwise multiple comparisons (see Supplementary Table 1) showed that the image noise of the CSO, the posterior fossa, and air was significantly different between all image reconstructions, with a gradual decrease from ASiR-V to higher DLIR strengths. The image noise of the thalamus, the PLIC, the M5 cortex, and the ICHs was significantly lower for DLIR-H and DLIR-M compared to ASiR-V, and for DLIR-H compared to DLIR-M; however, no significant difference was detected between DLIR-L and ASiR-V. Regarding CT attenuation, there was a slight, yet significant difference between ASiR-V and at least one of the DLIR strengths (see Table 2). Also, there was a significant difference between DLIR-L and DLIR-H, and DLIR-M and DLIR-H, for the CT attenuation of the PLIC and the M5 cortex.

**Table 2:** Comparison of quantitative image quality parameters for the noncontrast head CT series between ASiR-V and all three DLIR strength levels

	ASiR-V	DLIR-L	DLIR-M	DLIR-H	***P*** value
CT Attenuation (HU)					
Thalamic GM	33.77 ± 2.44*	34.13 ± 2.18	34.05 ± 2.03	33.95 ± 2.02	0.001
PLIC WM	24.69 ± 2.30*^ab^	25.33 ± 2.03^b^	25.43 ± 1.99^b^	25.64 ± 1.99	< 0.001
M5 GM	36.99 ± 2.10^ab^	36.63 ± 2.04^b^	36.50 ± 2.00^b^	36.22 ± 1.97	< 0.001
CSO WM	26.86 ± 2.17^b^	27.04 ± 2.00	27.08 ± 1.88	27.17 ± 1.81	0.002
Image noise (HU)					
Thalamic GM	5.51 ± 1.16^ab^	5.32 ± 1.11^ab^	4.39 ± 0.92^b^	3.46 ± 0.71	< 0.001
PLIC WM	5.74 ± 1.25^ab^	5.33 ± 1.02^ab^	4.31 ± 0.85^b^	3.23 ± 0.62	< 0.001
M5 GM	5.05 ± 1.04^ab^	4.87 ± 1.01^ab^	4.04 ± 0.83^b^	3.22 ± 0.72	< 0.001
CSO WM	5.46 ± 1.04*^ab^	5.10 ± 0.97^ab^	4.07 ± 0.78^b^	3.07 ± 0.58	< 0.001
PF (artifacts)	8.85 ± 1.39*^ab^	8.24 ± 1.02^ab^	7.12 ± 0.99^b^	5.94 ± 0.99	< 0.001
Air	6.83 ± 0.92*^ab^	4.75 ± 0.70^ab^	3.55 ± 0.60^b^	2.29 ± 0.50	< 0.001
ICH	6.01 ± 1.58^ab^	5.89 ± 1.62^ab^	5.00 ± 1.46^b^	4.23 ± 1.37	< 0.001
SNR					
Thalamic GM	6.40 ± 1.37^ab^	6.69 ± 1.43^ab^	8.09 ± 1.68^b^	10.22 ± 2.11	< 0.001
PLIC WM	4.50 ± 1.05*^ab^	4.93 ± 1.02^ab^	6.13 ± 1.29^b^	8.23 ± 1.68	< 0.001
M5 GM	7.61 ± 1.51^ab^	7.81 ± 1.52^ab^	9.38 ± 1.79^b^	11.74 ± 2.39	< 0.001
CSO WM	5.11 ± 1.11*^ab^	5.50 ± 1.14^ab^	6.89 ± 1.36^b^	9.15 ± 1.76	< 0.001
ICH	10.44 ± 3.75^ab^	10.73 ± 3.73^ab^	12.68 ± 4.55^b^	15.18 ± 5.72	< 0.001
CNR					
Thalamic GM-PLIC WM	1.65 ± 0.47^ab^	1.69 ± 0.44^ab^	2.03 ± 0.51^b^	2.53 ± 0.60	< 0.001
M5 GM-CSO WM	1.95 ± 0.50^ab^	1.95 ± 0.47^ab^	2.35 ± 0.54^b^	2.91 ± 0.65	< 0.001

*HU* Hounsfield units, *GM* gray matter, *WM* white matter, *PLIC* posterior limb of the internal capsule, *M5* M5 cortex region (lateral MCA territory) according to Alberta Stroke Program Early CT Score — ASPECTS, *CSO* centrum semiovale, *PF* posterior fossa, *ICH* intracranial hemorrhage, *SNR* signal-to-noise ratio, *CNR* contrast-to-noise ratio, *ASiR-V* adaptive statistical iterative reconstruction-Veo, *DLIR-L* deep learning–based image reconstruction low strength level, *DLIR-M* deep learning–based image reconstruction medium strength level, *DLIR-H* deep learning–based image reconstruction high strength level

Post hoc pairwise multiple comparisons procedure with the Dunn-Bonferroni test showed a statistically significant (*P* < 0.05) difference between means when compared with DLIR-L (*), DLIR-M (^a^), and DLIR-H (^b^)

The SNR of both white matter regions (PLIC and CSO) was significantly different between all image reconstructions, with a gradual increase from ASiR-V to DLIR-H of up to 82.9%. The SNR of both gray matter regions (thalamus and M5 cortex) was significantly higher for DLIR-H and DLIR-M compared to ASiR-V, of up to 59.7%; however, no significant difference was detected between DLIR-L and ASiR-V. The same was found for SNR of ICH where there was a gradual increase from ASiR-V to DLIR-H of 45.4%; however, there was no significant difference between DLIR-L and ASiR-V.

The CNR between the thalamus and the PLIC, as well as the CNR between the M5 cortex and the adjacent white matter of the CSO, was significantly higher for DLIR-H and DLIR-M compared to ASiR-V, by up to 53.3% and 49.2%, respectively. There was no significant difference between DLIR-L and ASiR-V.

### Qualitative image quality analysis

**Fig. 2:**
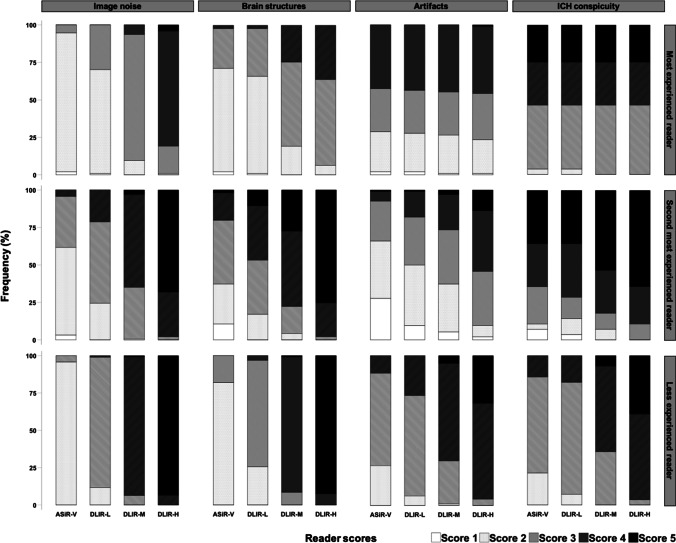
Stacked bar charts demonstrating reader scores as percentages in four parameters of qualitative image quality assessment (image noise, brain structures, artifacts, and intracranial hemorrhage (ICH) conspicuity) for ASiR-V, and all three DLIR strength levels (DLIR-L, DLIR-M, and DLIR-H). The distribution of scores for the DLIR reconstructions shifted towards higher scores compared to the distribution of scores for ASiR-V, for all parameters except for DLIR-L for ICH conspicuity for the most experienced reader, where the distribution of scores was the same as for ASiR-V. Furthermore, there was a tendency towards higher scores with increasing DLIR strength levels. The proportion of non-diagnostic CT series (i.e., “Score 1” for all image quality parameters as well as “Score 2” for ICH) was lower for DLIR-M and DLIR-H compared to ASiR-V, for all readers

**Fig. 3:**
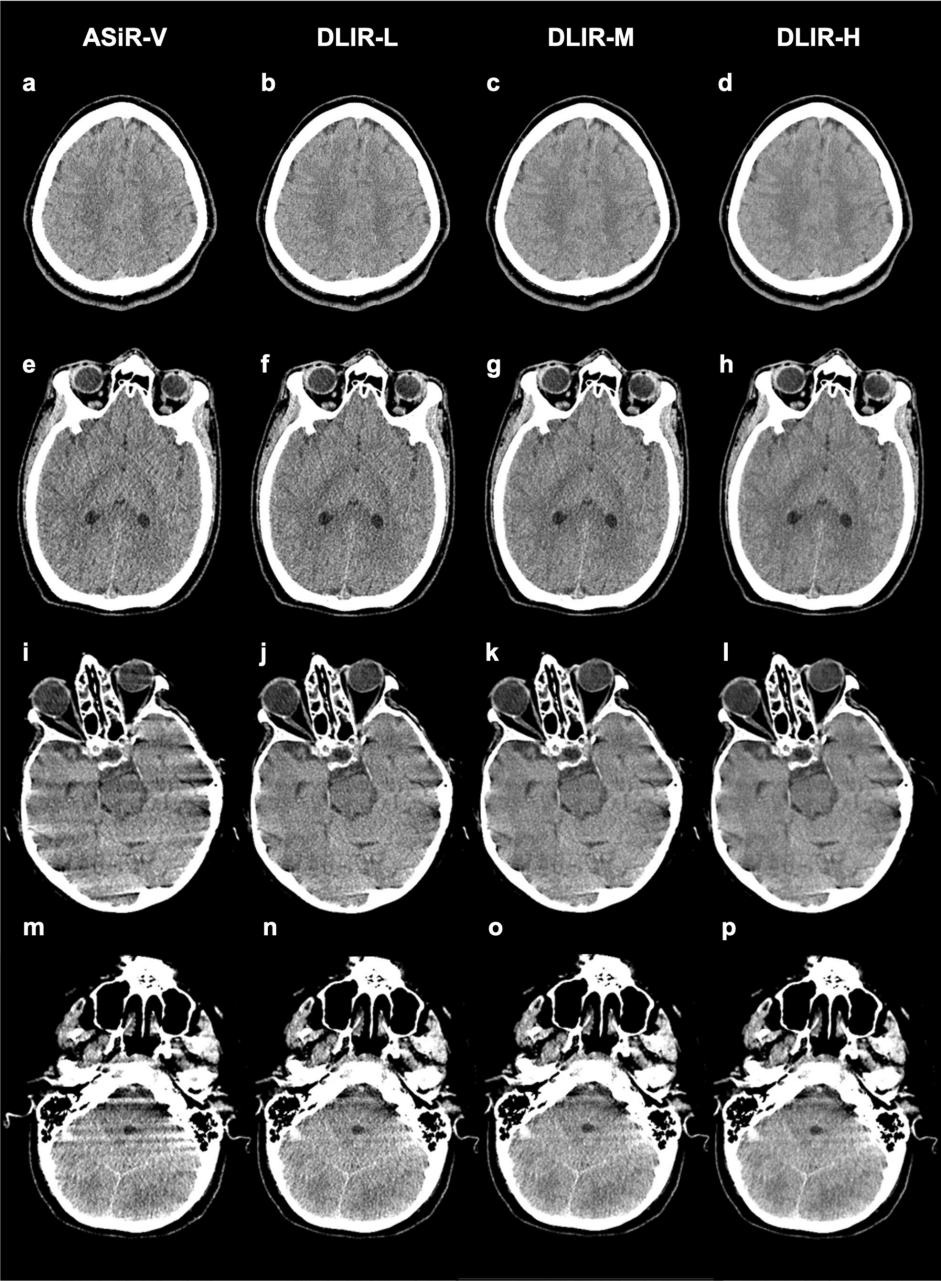
Axial noncontrast trauma head CT images reconstructed with ASiR-V (**a**, **e**, **i**, **m**), DLIR-L (**b**, **f**, **j**, **n**), DLIR-M (**c**, **g**, **k**, **o**), and DLIR-H (**d**, **h**, **l**, **p**). The first row of images (**a**–**d**) is of a 24-year-old male patient at the level of the centrum semiovale. The second row of images (**e**–**h**) is of a 41-year-old male patient at the level of the basal ganglia. The third row of images (**i**–**l**) is of a 67-year-old female at the level of the pons/interpeduncular cistern. The fourth row of images (**m**–**p**) is of a 36-year-old female at the level of the pons/interpetrous region. Readers graded image noise, brain structures, and artifacts higher for the DLIR reconstructions compared to ASiR-V. In addition, there was a tendency towards higher scores with increasing DLIR strength levels

**Fig. 4:**
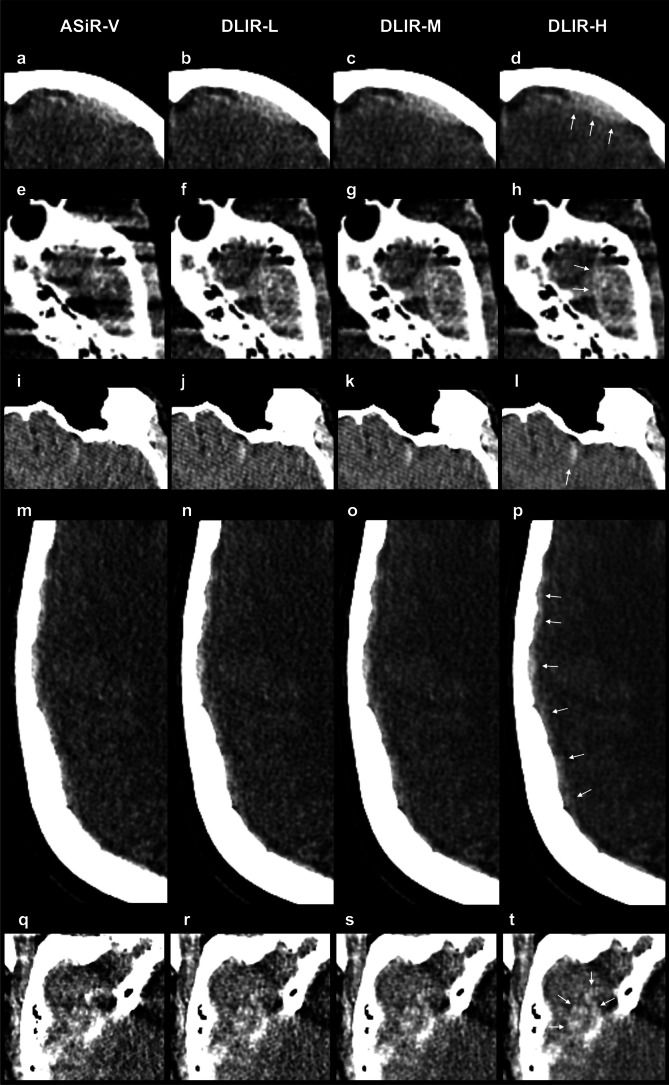
Axial noncontrast trauma head CT images with intracranial hemorrhages, reconstructed with ASiR-V (**a**, **e**, **i**, **m**,** q**), DLIR-L (**b**, **f**, **j**, **n**, **r**), DLIR-M (**c**, **g**, **k**, **o**, **s**), and DLIR-H (**d**, **h**, **l**, **p**, **t**). The first row of images (**a**–**d**) is of a 26-year-old female patient who was assaulted. A left frontal epidural hematoma was diagnosed (white arrows in image **d**). The second row of images (**e**–**h**) is of a 22-year-old male that was involved in a motor vehicle accident. At the level of the pons/interpetrous region, an epidural hematoma in the middle cranial fossa on the left side was diagnosed (white arrows in image **h**). The third row of images (**i**–**l**) is of a 33-year-old male, a pedestrian who was hit by a car. A left frontal subarachnoid hemorrhage was diagnosed (white arrow in image **l**). The fourth row of images (**m**–**p**) is of a 62-year-old female who fell at home. An acute subdural hematoma along the right cerebral convexity was diagnosed (white arrows in image **p**). The fifth row of images (**q–t**) is of a 69-year-old male who fell. Cerebral hemorrhagic contusions were diagnosed adjacent to the floor of the right middle cranial fossa (white arrows in image **t**), and all three readers graded the ASiR-V images as non-diagnostic (score 1 or 2) and the DLIR-H images as diagnostic (score > 2). Overall, regarding intracranial hemorrhage conspicuity, the readers graded fewer CT series as non-diagnostic (score 1 or 2) for DLIR-M and DLIR-H compared to ASiR-V and DLIR-L

The results from the qualitative image quality analysis are summarized in Table 3 and Fig. 2. The grading of the qualitative image quality parameters demonstrated a similar pattern when compared to the results of the quantitative analysis. For all three readers, the distribution of scores for the DLIR reconstructions shifted towards higher scores compared to the distribution of scores for ASiR-V, for all parameters except for DLIR-L for ICH conspicuity for the most experienced reader, where the distribution of scores was the same as for ASiR-V (Figs. 3 and 4). Additionally, the shift towards higher scores gradually increased with increasing DLIR strengths for all parameters and readers, except between DLIR-M and DLIR-H for ICH for the most experienced reader where the distribution of the scores was the same. For the less experienced reader, the results from the pairwise comparisons (Supplementary Table 2) showed a significant difference between all image reconstructions for all parameters except between ASiR-V and DLIR-L, for ICH conspicuity. For the second most experienced reader, the pairwise comparisons demonstrated a similar result when compared to the less experienced reader with the addition that no significant difference was detected between DLIR-L and DLIR-M for artifacts and ASiR-V and DLIR-M, and DLIR-L and DLIR-M, and DLIR-M and DLIR-H, for ICH conspicuity. For the most experienced reader, for image noise, the pairwise comparisons showed significantly different scores between all image reconstructions except between ASiR-V and DLIR-L. For the same reader for brain structures, there was a significant difference between all image reconstructions, except between ASiR-V and DLIR-L, and DLIR-M and DLIR-H. No significant differences were detected between the reconstructions for artifacts and ICH scores in the most experienced reader.

**Table 3:** Comparison of qualitative image quality scores for the noncontrast head CT series between ASiR-V and all three DLIR strength levels

Reader and parameters (distribution of scores 1/2/3/4/5 given as percentages)		ASiR-V	DLIR-L	DLIR-M	DLIR-H	*P* value
**Most experienced reader**	Image noise	2.1/92.6/5.3/0/0^ab^	1.1/69.1/29.8/0/0^ab^	0/9.6/84.0/6.4/0^b^	0/1.1/18.1/76.6/4.3	< 0.001
	Brain structures	2.1/69.1/26.6/2.1/0^ab^	1.1/64.9/31.9/2.1/0^ab^	0/19.1/56.4/24.5/0	0/6.4/57.4/36.2/0	< 0.001
	Artifacts	2.1/26.6/28.7/42.6/0	2.1/25.5/28.7/43.6/0	1.1/25.5/28.7/44.7/0	1.1/22.3/30.9/44.7/1.1	< 0.001
	ICH conspicuity	0/3.6/42.9/28.6/25.0	0/3.6/42.9/28.6/25.0	0/0/46.4/28.6/25.0	0/0/46.4/28.6/25.0	ns (0.392)
**Second most experienced reader**	Image noise	3.2/58.5/34.0/4.3/0*^ab^	0/24.5/54.3/21.3/0^ab^	0/1.1/34.0/61.7/3.2^b^	0/0/2.1/29.8/68.1	< 0.001
	Brain structures	10.6/26.6/42.6/18.1/2.1*^ab^	0/17.0/36.2/36.2/10.6^ab^	0/4.3/18.1/50.0/27.7^b^	0/0/2.1/22.3/75.5	< 0.001
	Artifacts	27.7/38.3/26.6/6.4/1.1*^ab^	9.6/40.4/31.9/17.0/1.1^b^	5.3/31.9/36.2/23.4/3.2^b^	2.1/7.4/36.2/40.4/13.8	< 0.001
	ICH conspicuity	7.1/3.6/25.0/28.6/35.7^b^	3.6/10.7/14.3/35.7/35.7^b^	0/7.1/10.7/28.6/53.6	0/0/10.7/25.0/64.3	< 0.001
**Less experienced reader**	Image noise	0/95.7/4.3/0/0*^ab^	0/11.7/87.2/1.1/0^ab^	0/0/6.4/92.6/1.1^b^	0/0/0/6.4/93.6	< 0.001
	Brain structures	0/81.9/18.1/0/0*^ab^	0/25.5/71.3/3.2/0^ab^	0/0/8.5/90.4/1.1^b^	0/0/0/7.4/92.6	< 0.001
	Artifacts	0/26.6/60.6/12.8/0*^ab^	0/6.4/66.0/27.7/0^ab^	0/1.1/28.7/63.8/6.4^b^	0/0/4.3/63.8/31.9	< 0.001
	ICH conspicuity	0/21.4/64.3/14.3/0^ab^	0/7.1/75.0/17.9/0^ab^	0/0/35.7/57.1/7.1^b^	0/0/3.6/57.1/39.3	< 0.001

*ICH* intracranial hemorrhage, *ASiR-V* adaptive statistical iterative reconstruction-Veo, *DLIR-L* deep learning–based image reconstruction low strength level, *DLIR-M* deep learning–based image reconstruction medium strength level, *DLIR-H* deep learning–based image reconstruction high strength level, *ns* not significant

Post hoc pairwise multiple comparisons procedure with the Dunn-Bonferroni test showed a statistically significant (*P* < 0.05) difference between means when compared with DLIR-L (*), DLIR-M (^a^), and DLIR-H (^b^)

The proportion of non-diagnostic scores (i.e., “score 1” for all parameters as well as “score 2” for ICH) decreased with increasing DLIR strengths for all readers (Table 3). Compared to ASiR-V, the reconstructions DLIR-M and DLIR-H demonstrated smaller percentages of non-diagnostic scores for all readers. Notably, regarding ICH conspicuity, fewer CT series were regarded as non-diagnostic (score 1 or 2) for DLIR-M and DLIR-H compared to ASiR-V and DLIR-L, and none was graded as non-diagnostic for DLIR-H.

The inter-reader agreement between the most experienced and the less experienced reader was moderate for image noise (*κ*_w_ = 0.41), fair for brain structures (*κ*_w_ = 0.25), and poor for artifacts and ICH (*κ*_w_ = 0.13 and 0.09, respectively). The agreement between the less experienced and the second most experienced reader was good for image noise (*κ*_w_ = 0.67), moderate for brain structures (*κ*_w_ = 0.48), and fair for artifacts and ICH (both *κ*_w_ = 0.24). The agreement between the most experienced and the second most experienced reader was fair for image noise (*κ*_w_ = 0.36), poor for brain structures (*κ*_w_ = 0.12), and fair for artifacts and ICH (*κ*_w_ = 0.21 and 0.29, respectively).

## Discussion

We compared the image quality between a DL-based (TrueFidelity) and an IR-based (ASiR-V) image reconstruction algorithm for trauma head CT. We have demonstrated that TrueFidelity outperforms ASiR-V regarding both quantitative and qualitative image quality parameters. The image quality increased with higher DLIR strengths. In particular, DLIR-M and DLIR-H had significantly lower image noise levels and higher SNR and CNR compared to ASiR-V. DLIR-M and DLIR-H also received higher reader scores compared to ASiR-V and DLIR-L. Furthermore, the proportion of non-diagnostic CT series was lower for DLIR-M and DLIR-H when compared to ASiR-V, for all readers. The improvement in qualitative image quality provided by DLIR was greater for the second most and the less experienced reader than for the most experienced reader.

These findings are in line with the current literature which shows that DLIR algorithms achieve superior image quality for head CT compared to IR-based algorithms [23–25, 29]. We confirm the findings from a similar study by Kim et al. [23]. However, they included all patients undergoing a noncontrast head CT, no matter the indication, and excluded pathology. We showed that the superior image quality of TrueFidelity compared to ASiR-V also applies to trauma patients with the potential of improving diagnostic performance regarding ICH. Furthermore, we recruited three readers with different levels of experience, as opposed to the study by Kim et al. in which two experienced neuroradiologists performed the qualitative image quality analysis. Not only were the readers in our study blinded to each other’s evaluations, but also to each other’s identity. Another strength with our study is that each reader received an individual training session before initiating image evaluation. Additionally, although debatable, we consider our approach of presenting the ordinal data (reader scores) as percentages a more appropriate measure than the mean. We also included a larger number of patients in our study.

The study by Sun et al. was performed on a pediatric population and even though trauma cases were included in this study the patient cohort was quite heterogenous and also included patients with convulsion or mental symptoms or cases where emergency doctors requested a CT scan to exclude intracranial pathology [24]. We have focused on the trauma patient subset exclusively and thereby showed that DLIR can successfully be implemented for head CT on trauma patients presenting to a level 1 trauma unit. Additional strengths with our study are that we almost had the double sample size compared to Sun et al.

In a retrospective study, Wong et al. [29] developed a novel DL-based CT image denoising method where the model was trained on noncontrast head CT in patients with acute ischemic stroke. Each CT scan was unique regarding imaging protocol, scanner vendor and model, radiation dose, etc. A limitation of their study is that they only included three cases in the assessment of stroke visualization and that they did not quantify the direct visual comparisons. Furthermore, the visual comparisons were not performed by radiologists. Also, the denoising performance assessment was only performed using the top 30 images of the supratentorial region. The strengths with our study compared to that of Wong et al. are that we included quantitative image quality parameters on several anatomical levels and also evaluated qualitative parameters in a quantized and standardized manner. Furthermore, our qualitative image quality assessment was performed by end-users.

Our study confirms the findings by Oostveen et al., who detected lower image noise and improved gray-white matter differentiation of DLIR compared to IR-based image reconstruction algorithms for noncontrast head CT [25]. However, they included patients who underwent a head CT scan for various indications and did not incorporate intracranial pathology in their image quality assessment. Also, our sample size was nearly twice as large.

Our results are also in line with a review by Arndt et al. who evaluated DLIR phantom and body studies and concluded that DLIR algorithms improve image quality with the potential for radiation dose reduction [30]. Another review by Zhang et al. confirms that DLIR preserves image quality better at low doses compared to other image reconstruction techniques [20]. However, the nonlinear properties of DLIR algorithms can occasionally cause complex and unanticipated effects on image quality parameters. One study has reported that even though DLIR algorithms achieve less noise than FBP or IR, the spatial resolution can become degraded, especially with a decreasing radiation dose of the training images for the DLIR algorithm [31]. Furthermore, a phantom study by Solomon et al. showed superior low-contrast resolution of FBP compared to both ASiR-V and TrueFidelity and the low-contrast resolution decreased with increasing ASiR-V and DLIR strengths [32]. Another study by Jensen et al. reported blurring of small abdominal lesions that increases with higher DLIR strengths [33], rather analogous to what has been observed with higher IR strengths [14–16]. We did not find any degradation of the image quality at higher DLIR strengths. We detected subtle, yet statistically significant differences in CT attenuation between the reconstructions (Table 2). A further analysis of the mean absolute HU differences as pairwise differences between all possible combinations of the four image reconstruction types (Supplementary Table 3) showed that the HU differences were slightly greater between ASiR-V and any DLIR strength level than between any possible combination of DLIR strength pairs, for all four brain regions. Additionally, a greater number of cases with statistically significant mean absolute HU differences were detected between pairs where one of the reconstruction types in one or both pairs was ASiR-V (37 cases), compared to if it was DLIR-L (16 cases), DLIR-M (15 cases), or DLIR-H (14 cases). These findings indicate that DLIR might cause minimal alterations in the attenuation values (in our case mean absolute HU differences of up to 1.03 HU). The clinical consequences of this finding are most likely insignificant.

There was a substantial variability in inter-reader agreement. This could partially be due to the implementation of a 5-grade scoring system. A scoring system with fewer scoring levels could have resulted in a higher inter-reader agreement, at the expense of less specific qualitative image scores. Another contributing factor to the variability in *κ*_w_-values could be that the scores for artifacts and ICH conspicuity for the most experienced reader were relatively uniform between the different image reconstructions compared to the second most and the less experienced readers. This could also indicate that the superior image quality of DLIR did not influence the diagnostic confidence of the most experienced reader to the same degree as it did for the second most and the less experienced readers. Further studies are needed to evaluate if DLIR increases the diagnostic confidence for trauma head CT to a greater extent in less experienced compared to more experienced readers. If this is the case, it could lead to improved acute diagnostic accuracy for head CT for trauma patients at our trauma unit as the preliminary interpretation of the trauma CT usually is performed by our radiology residents (which is probably true for many trauma units during on call hours).

There are several limitations of our study. First, patients were evaluated retrospectively at a single level 1 trauma center. Second, the study did not directly assess diagnostic performance, which is necessary to fully appreciate the potential clinical benefits of DLIR. Third, images were obtained by a standard dose CT protocol. Hence, the radiation dose reduction potential of DLIR could not be directly determined. Fourth, assessment of normal anatomical structures was also performed in cases with ICH. Even though the evaluation in these cases was performed on the unaffected contralateral hemisphere, confounding effects on image evaluation due to the pathology cannot be fully excluded. Fifth, even though a spontaneous intracerebral hemorrhage is not a trauma-related finding per se, we decided to include this ICH type in the study as it is found in a small fraction of the trauma patients that present to our trauma unit and therefore mirrors the clinical scenario. Sixth, the CT scans were obtained with our standard trauma CT protocol with a slice thickness of 0.625 mm with 0.3125 mm overlap. However, the applied version of TrueFidelity on the raw data did not offer overlapping reconstructions. We are confident that this difference did not significantly affect the image quality comparison between ASiR-V and DLIR. If anything, the overlapping slices could potentially improve the image quality of ASiR-V by increasing the detection of small lesions [34]. We did not alter our standard trauma CT protocol by removing the slice overlap because we wanted to reflect the clinical scenario at our department as far as possible as this project also served the purpose as quality control for a novel trauma CT protocol. Seventh, we only evaluated how DLIR performs compared to ASiR-V, an IR technique based on hybrid/adapted-IR. We did not evaluate how DLIR performs compared to full/model-based IR techniques (MBIR). A phantom study by Higaki et al. has demonstrated that the image quality achieved by DLIR at low radiation doses outperforms that achieved by MBIR, but that MBIR outperforms DLIR at high radiation doses [35]. In a review, Nakamura et al. stated that DLIR may enhance the detection of low-contrast lesions compared to MBIR. They also indicated that MBIR enhances the detection of high-contrast lesions at high radiation doses compared to DLIR [36]. Further studies are needed to evaluate the clinical implications of these findings. Eighth, the calculated SNRs in our study do not represent true SNRs. True signal is the output signal of the detector, which, for energy-integrating detectors, is proportional to the integrated energy levels of all received photons [37]. The HU in turn is the linear transformation of the measured linear attenuation coefficient [38]. It would have been sufficient only to present the image noise. However, SNR, as defined in this article, is a frequently encountered quantitative CT image quality parameter in the literature, probably recognizable by many readers and easily related to.

Our findings are in line with the current abovementioned literature and extends the superior image quality of DLIR for head CT to the trauma setting as well. Further studies are needed to evaluate the performance of DLIR for other anatomical locations and spectrum of traumatic injuries as well as to determine if DLIR increases the diagnostic confidence to a higher degree for less experienced than for more experienced readers. A next step after that would be to determine the degree of radiation dose reduction that DLIR allows while maintaining diagnostic image quality in trauma patients.

In conclusion, the image quality of trauma head CT series reconstructed with DLIR outperformed CT series reconstructed with ASiR-V. In particular, DLIR-M and DLIR-H demonstrated significantly improved image quality and a lower proportion of non-diagnostic images. The qualitative image quality improvement provided by DLIR was more evident for the second most and the less experienced readers compared to the most experienced reader.

## Supplementary Information

Below is the link to the electronic supplementary material.

Supplementary file1 (DOCX 22.1 KB)

Supplementary file2 (DOCX 29.4 KB)

## Abbreviations

AI: Artificial intelligence
ANOVA: Analysis of variance
ASiR-V: Adaptive statistical iterative reconstruction-Veo
ASPECTS: Alberta Stroke Program Early CT Score
CNR: Contrast-to-noise ratio
CSO: Centrum semiovale
CT: Computed tomography
CTDIvol: Volume CT dose index
DL: Deep learning
DLIR: Deep learning–based image reconstruction
DLIR-L: Deep learning–based image reconstruction low
DLIR-M: Deep learning–based image reconstruction medium
DLIR-H: Deep learning–based image reconstruction high
DLP: Dose-length product
FBP: Filtered back projection
GE: General Electric
HU: Hounsfield unit
ICH: Intracranial hemorrhage
IR: Iterative reconstruction
MCA: Middle cerebral artery
ML: Machine learning
PACS: Picture archiving and communication system
PLIC: Posterior limb of the internal capsule
ROI: Region of interest
SD: Standard deviation
SNR: Signal-to-noise ratio

## Declarations

### Guarantor

The scientific guarantor of this publication is Koskinen Seppo K.

### Statistics* and biometry*

No complex statistical methods were necessary for this paper.

### Study subjects or cohorts overlap

Study subjects have not been previously reported.

### Methodology

• Retrospective

• Diagnostic study (indirect assessment of diagnostic performance)

• Performed at one institution
