# Supplementary material for: Deep learning versus iterative image reconstruction algorithm for head CT in trauma
Source: Emerg Radiol. 2022 Jan 5;29(2):339–52. doi: 10.1007/s10140-021-02012-2 (PMC8917108; doi:10.1007/s10140-021-02012-2)
Supplement: Supplementary file 1 — Supplementary file1 (DOCX 22.1 KB) [file 10140_2021_2012_MOESM1_ESM.docx]

**Supplementary Table 3.** Mean CT attenuation pairwise differences (presented as absolute values) between all combinations of the four image reconstruction types.

|  | **ASiR-V** – **DLIR-L** | **ASiR-V** –**DLIR-M** | **ASiR-V** –**DLIR-H** | **DLIR-L** –**DLIR-M** | **DLIR-L** –**DLIR-H** | **DLIR-M** –**DLIR-H** | ***P* value** |
| --- | --- | --- | --- | --- | --- | --- | --- |
| **ΔCT ATTENUATION (HU)** |  |  |  |  |  |  |  |
| Thalamic GM | 0.86±0.61^bcd^ | 0.85±0.61^bcd^ | 0.80±0.61^bcd^ | 0.33±0.24 | 0.36±0.33 | 0.27±0.23 | < 0.001 |
| PLIC WM | 0.79±0.69^abd^ | 0.85±0.72^abcd^ | 1.03±0.77^bcd^ | 0.28±0.25^c^ | 0.44±0.29 | 0.33±0.21 | < 0.001 |
| M5 GM | 0.73±0.71^abd^ | 0.73±0.72^abd^ | 1.00±0.82^bcd^ | 0.35±0.25^c^ | 0.54±0.38^d^ | 0.39±0.33 | < 0.001 |
| CSO WM | 0.52±0.45^abd^ | 0.57±0.48^abd^ | 0.75±0.53^bcd^ | 0.27±0.25 | 0.40±0.31^d^ | 0.29±0.25 | < 0.001 |

*ΔCT ATTENUATION (HU)* mean absolute CT attenuation difference in Hounsfield units, *GM* gray matter, *WM* white matter, *PLIC* posterior limb of the internal capsule, *M5* M5 cortex region (lateral MCA territory) according to Alberta Stroke Program Early CT Score – ASPECTS, *CSO* centrum semiovale, *ASiR-V* adaptive statistical iterative reconstruction-Veo, *DLIR-L* deep learning-based image reconstruction low strength level, *DLIR-M* deep learning-based image reconstruction medium strength level, *DLIR-H* deep learning-based image reconstruction high strength level

Post hoc pairwise multiple comparisons procedure with the Dunn-Bonferroni test showed a statistically significant (*P* <0.05) difference between means when compared with pairwise difference group ‘ASiR-V–DLIR-M’ (*), ‘ASiR-V–DLIR-H’ (^a^), ‘DLIR-L–DLIR-M’ (^b^), ‘DLIR-L–DLIR-H’ (^c^), and ‘DLIR-M–DLIR-H’ (^d^)
