# Supplementary material for: Deep learning versus iterative image reconstruction algorithm for head CT in trauma
Source: Emerg Radiol. 2022 Jan 5;29(2):339–52. doi: 10.1007/s10140-021-02012-2 (PMC8917108; doi:10.1007/s10140-021-02012-2)
Supplement: Supplementary file 2 — Supplementary file2 (DOCX 29.4 KB) [file 10140_2021_2012_MOESM2_ESM.docx]

**Supplementary Table 1.** Post hoc pairwise p-values (Bonferroni adjusted) from the quantitative image quality analysis, for all reconstruction type pairs

|  | **ASiR-V vs. DLIR-L** | **ASiR-V vs. DLIR-M** | **ASiR-V vs. DLIR-H** | **DLIR-L vs. DLIR-M** | **DLIR-L vs. DLIR-H** | **DLIR-M vs. DLIR-H** |
| --- | --- | --- | --- | --- | --- | --- |
| **CT ATTENUATION** |  |  |  |  |  |  |
| Thalamic GM | **0.002** | 0.084 | 1 | 1 | 0.052 | 0.898 |
| PLIC WM | **< 0.001** | **< 0.001** | **< 0.001** | 1 | **< 0.001** | **0.002** |
| M5 GM | 0.066 | **< 0.001** | **< 0.001** | 0.078 | **< 0.001** | **0.005** |
| CSO WM | 0.947 | 0.154 | **0.001** | 1 | 0.098 | 0.682 |
| **IMAGE NOISE** |  |  |  |  |  |  |
| Thalamic GM | 0.763 | **< 0.001** | **< 0.001** | **< 0.001** | **< 0.001** | **< 0.001** |
| PLIC WM | 0.078 | **< 0.001** | **< 0.001** | **< 0.001** | **< 0.001** | **< 0.001** |
| M5 GM | 1 | **< 0.001** | **< 0.001** | **< 0.001** | **< 0.001** | **< 0.001** |
| CSO WM | **0.028** | **< 0.001** | **< 0.001** | **< 0.001** | **< 0.001** | **< 0.001** |
| PF (artifacts) | **< 0.001** | **< 0.001** | **< 0.001** | **< 0.001** | **< 0.001** | **< 0.001** |
| Air | **< 0.001** | **< 0.001** | **< 0.001** | **< 0.001** | **< 0.001** | **< 0.001** |
| ICH | 1 | **< 0.001** | **< 0.001** | **< 0.001** | **< 0.001** | **0.016** |
| **SNR** |  |  |  |  |  |  |
| Thalamic GM | 0.479 | **< 0.001** | **< 0.001** | **< 0.001** | **< 0.001** | **< 0.001** |
| PLIC WM | **0.009** | **< 0.001** | **< 0.001** | **< 0.001** | **< 0.001** | **< 0.001** |
| M5 GM | 1 | **< 0.001** | **< 0.001** | **< 0.001** | **< 0.001** | **< 0.001** |
| CSO WM | **0.006** | **< 0.001** | **< 0.001** | **< 0.001** | **< 0.001** | **< 0.001** |
| ICH | 1 | **< 0.001** | **< 0.001** | **< 0.001** | **< 0.001** | **0.016** |
| **CNR** |  |  |  |  |  |  |
| Thalamic GM-PLIC WM | 1 | **< 0.001** | **< 0.001** | **< 0.001** | **< 0.001** | **< 0.001** |
| M5 GM-CSO WM | 1 | **< 0.001** | **< 0.001** | **< 0.001** | **< 0.001** | **< 0.001** |

*GM* gray matter, *WM* white matter, *PLIC* posterior limb of the internal capsule, *M5* M5 cortex region (lateral MCA territory) according to Alberta Stroke Program Early CT Score – ASPECTS, *CSO* centrum semiovale, *PF* posterior fossa, *ICH* intracranial hemorrhage, *SNR* signal-to-noise ratio, *CNR* contrast-to-noise ratio, *ASiR-V* adaptive statistical iterative reconstruction-Veo, *DLIR-L* deep learning-based image reconstruction low strength level, *DLIR-M* deep learning-based image reconstruction medium strength level, *DLIR-H* deep learning-based image reconstruction high strength level

Bold type indicates statistical significance

**Supplementary Table 2.**  Post hoc pairwise p-values (Bonferroni adjusted) from the qualitative image quality analysis, for all reconstruction type pairs

|  | **ASiR-V vs. DLIR-L** | **ASiR-V vs. DLIR-M** | **ASiR-V vs. DLIR-H** | **DLIR-L vs. DLIR-M** | **DLIR-L vs. DLIR-H** | **DLIR-M vs. DLIR-H** |
| --- | --- | --- | --- | --- | --- | --- |
| **Most experienced reader** |  |  |  |  |  |  |
| Image noise | 0.220 | **< 0.001** | **< 0.001** | **< 0.001** | **< 0.001** | **< 0.001** |
| Brain structures | 1 | **< 0.001** | **< 0.001** | **< 0.001** | **< 0.001** | 0.722 |
| Artifacts | 1 | 1 | 1 | 1 | 1 | 1 |
| ICH conspicuity | ns | ns | ns | ns | ns | ns |
| **Second most experienced reader** |  |  |  |  |  |  |
| Image noise | **0.002** | **< 0.001** | **< 0.001** | **< 0.001** | **< 0.001** | **< 0.001** |
| Brain structures | **0.002** | **< 0.001** | **< 0.001** | **< 0.001** | **< 0.001** | **< 0.001** |
| Artifacts | **0.004** | **< 0.001** | **< 0.001** | 0.098 | **< 0.001** | **< 0.001** |
| ICH conspicuity | 1 | 0.156 | **0.005** | 0.261 | **0.010** | 1 |
| **Less experienced reader** |  |  |  |  |  |  |
| Image noise | **< 0.001** | **< 0.001** | **< 0.001** | **< 0.001** | **< 0.001** | **< 0.001** |
| Brain structures | **0.008** | **< 0.001** | **< 0.001** | **< 0.001** | **< 0.001** | **< 0.001** |
| Artifacts | **0.044** | **< 0.001** | **< 0.001** | **< 0.001** | **< 0.001** | **< 0.001** |
| ICH conspicuity | 1 | **0.004** | **< 0.001** | **0.031** | **< 0.001** | **0.043** |

*ICH* intracranial hemorrhage, *ASiR-V* adaptive statistical iterative reconstruction-Veo, *DLIR-L* deep learning-based image reconstruction low strength level, *DLIR-M* deep learning-based image reconstruction medium strength level, *DLIR-H* deep learning-based image reconstruction high strength level, *ns* not significant (post hoc test not performed due to nonsignificant repeated-measures ANOVA/Friedman test)

Bold type indicates statistical significance
